# A Novel Cis-Regulatory lncRNA, *Kalnc2*, Downregulates *Kalrn* Protein-Coding Transcripts in Mouse Neuronal Cells

**DOI:** 10.3390/ncrna9010007

**Published:** 2023-01-13

**Authors:** Muneesh Pal, Divya Chaubey, Mohit Tanwar, Beena Pillai

**Affiliations:** 1 CSIR-Institute of Genomics and Integrative Biology, Delhi 110025, India; 2 Academy of Scientific and Innovative Research (AcSIR), Ghaziabad 201002, India

**Keywords:** *Kalrn*, lncRNA, *durga*, neuron, dendrite, chromatin

## Abstract

The *KALRN* gene encodes several multi-domain protein isoforms that localize to neuronal synapses, conferring the ability to grow and retract dendritic spines and shaping axonal outgrowth, dendrite morphology, and dendritic spine re-modeling. The *KALRN* genomic locus is implicated in several neurodevelopmental and neuropsychiatric diseases, including autism, schizophrenia, bipolar disease, and intellectual disability. We have previously shown that a novel brain-specific long non-coding RNA (lncRNA) arising from the 5′ end of the *kalrna* gene, called *durga*, regulates neuronal morphology in zebrafish. Here, we characterized mammalian *Kalrn* loci, annotating and experimentally validating multiple novel non-coding RNAs, including linear and circular variants. Comparing the mouse and human loci, we show that certain non-coding RNAs and *Kalrn* protein-coding isoforms arising from the locus show similar expression dynamics during development. In humans, mice, and zebrafish, the 5′ end of the *Kalrn* locus gives rise to a chromatin-associated lncRNA that is present in adult ovaries, besides being expressed during brain development and enriched in certain regions of the adult brain. Ectopic expression of this lncRNA led to the downregulation of all the major *Kalrn* mRNA isoforms. We propose that this lncRNA arising from the 5′ end of the *Kalrn* locus is functionally the mammalian ortholog of zebrafish lncRNA *durga*.

## 1. Introduction:

The *KALRN* gene is located on chromosome three of the human genome and gives rise to the Dbl family Guanine nucleotide exchange factor protein called Kalirin. It was originally identified as P-CIP10 in a screen for protein-protein interactions [1] and subsequently named Kalirin [2]. Later, it was also described as an interacting partner of Huntingtin-associated protein1 and called HAPIP [3]. It is expressed selectively in the brain and central nervous system, while highly similar proteins like DUO and TRIO show expression in other tissues too [4]. Kalirin protein consists of many protein-protein interaction domains that allow it to interact with several partners in the dendritic spine. Most of its functions are dependent on the RAC and Rho GTPase functions, which result in the release of GDP from RAC and Rho and activation of GTP signaling in the cell. For instance, the Kalirin-7 (Kal7) isoform localizes to the dendritic spines and interacts with PDZ domain-containing proteins using a specific region in its C-terminal to regulate dendritic morphogenesis [5]. Kalirin protein isoforms are known to affect the formation of dendritic spines, dendritic arborization, axonal growth, synapse formation, and plasticity [4,6,7].

Mutations in the *KALRN* gene that are deleterious to protein function are associated with several neurodevelopmental diseases like autism and intellectual disability, as well as neuropsychiatric diseases like schizophrenia [8,9,10,11,12]. Intronic variants that do not change the protein product but result in altered expression have been linked to addiction and reward anticipation [13]. *KALRN* expression was also found to be reduced in post-mortem Alzheimer’s brain samples. Overall, the disease phenotypes seen in humans agree well with the loss of expression or function of Kalirin protein and resultant behavioral effects seen in mouse models. An interesting complication is the disparate effects of the Rac and Rho GEF domains: mutations in the Rac GEF domain reduce RAC signaling, resulting in reduced neuronal branching and spine density, and lead to a decrease in cortical thickness, while a mutation in the Rho GEF domain had similar effects in spite of activating Rho GTP signaling [7,14].

The multi-function Kalirin protein exerts its diverse effects through its many isoforms. The locus consists of 60 exons in humans, which give rise to 52 transcript isoforms. The regulated expression of *Kalrn* isoforms is thought to be driven by differential promoter usage, although these promoters are yet to be characterized in detail. The transcript isoforms can be clustered into two major groups: One group of relatively short isoforms, mostly including the exons at the 5′ end, includes the Kal7 isoform that has been studied extensively in mice and cell culture models. Kal7 knockout also resulted in schizophrenia-like effects in adolescent mice [15]. The Kal7 isoform includes the RacGEF domain but not the RhoGEF domain. Restoring levels of Kal7 can reverse the Alzheimer’s-like effects shown by mice with reduced Kal7 [16,17]. Besides the catalytic RacGEF domain that is critical to its function, it also contains a C-terminal PDZ domain, which is thought to target it to post-synaptic regions, and this facilitates its interactions with other proteins in this subcellular compartment. The longer isoforms, which include the RhoGEF domain, form a distinct group comprising Kalirin-9 (Kal9) and Kalirin-12 (Kal12), which play important roles during neuronal development [18,19,20]. Although it is clear from mutations linked to diseases and phenotypes in mouse models that the correct spatio-temporal expression of Kalirin isoforms is as critical as the final protein product itself, there is relatively little information on the regulation of the genomic locus.

Long non-coding RNAs are RNA transcripts more than 200 nts in length that do not code for functional proteins but may exert a variety of regulatory roles ranging from miRNA sponging and clearance to providing scaffolds for assembling ribo-nucleoprotein complexes and tethers for chromatin modifiers [21,22]. In zebrafish, we have reported that a maternally inherited long non-coding RNA, named durga, is the transcript expressed earliest in the developmental time frame, from the *Kalrn* locus. The non-coding RNA partially overlaps with the first exon of protein-coding *Kalrn* isoforms and is expressed during an early developmental window and later in certain regions of the adult zebrafish brain [23].

To identify similar non-coding RNAs from the mammalian *Kalrn* locus, we carefully annotated the transcripts reported in transcriptomic studies. By combining data from genome and transcriptome data repositories, we built a detailed map of the non-coding transcripts arising from the mouse *Kalrn* locus, including seven linear and one circular RNA. The linear RNAs are henceforth called *Kalnc1* to *7* in the order of their positions from the 5′ to 3′ ends of the mouse *Kalrn* gene. *Kalnc2* arises from the 5′ end close to the three known alternative start sites of *Kalrn* mRNA. During the in vitro differentiation of mouse primary cortical neurons, *Kalnc2* was down-regulated in mature neurons. We used RNA from post-mortem human tissues and a human neuronal cell line to show that the human *KALRN* locus also gives rise to a potentially non-coding transcript arising from the 5′ end of the *KALRN* gene, which shares short stretches of sequence similarity and functional features like chromatin association with *Kalnc2* and shows selective enrichment in immature neurons. In a mouse neuronal cell line, *Kalnc2*, overexpressed, leads to the downregulation of all major protein-coding transcripts of the *Kalrn* gene.

## 2. Results

**Figure 1:**
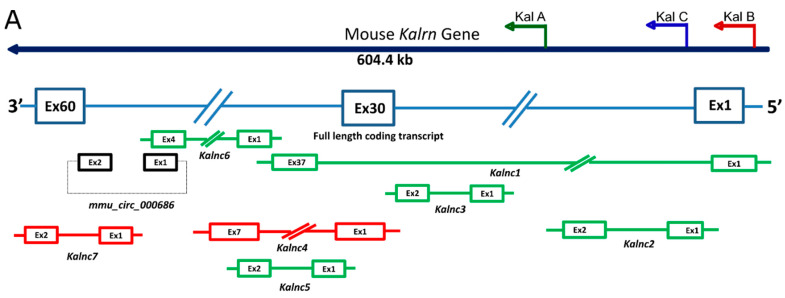

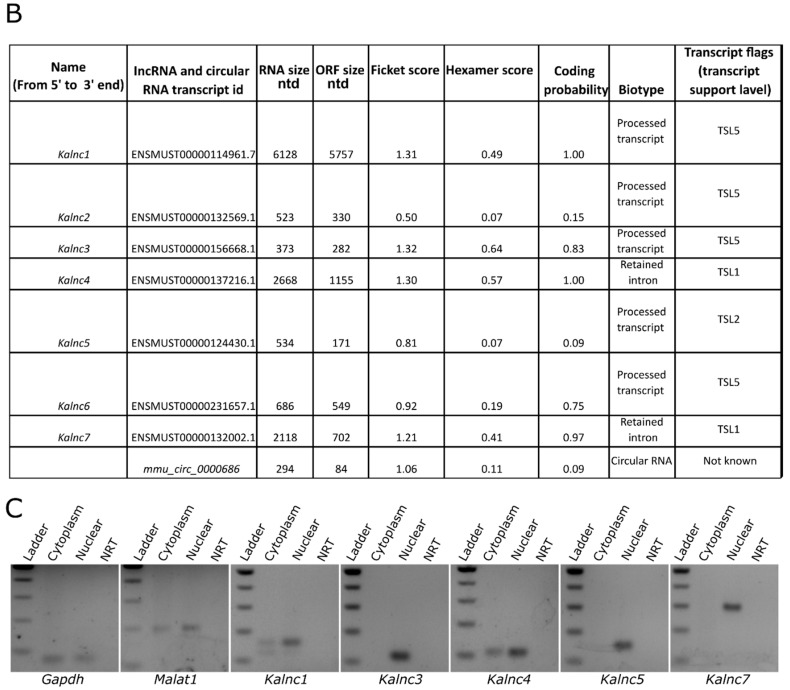
Schematic illustration and coding potential prediction of mouse *Kalrn* gene locus noncoding transcripts. (**A**) A schematic illustration of mouse *Kalrn* long noncoding transcripts (lncRNAs) was made according to the Ensembl database; blue: coding transcripts, green: processed transcripts, red: retained introns, black: circular RNA. Noncoding (NC) transcripts were named in the 5′ end (corresponding to the quantitatively major 5′ end called ‘C’) to 3′ end direction from *Kalnc1* to *Kalnc7*. (**B**) Coding potential was measured by CPAT software [26]. Transcript flags, or transcript support level (TSL), are a method to highlight the well-supported and poorly-supported transcript models. The method relies on the primary data that can support full-length transcript structure: mRNA and EST alignment supplied by UCSC and Ensembl. TSL1—all splice junctions of the transcript are supported by at least one non-suspect mRNA. TSL2—the best supporting mRNA is flagged as the suspect, or the support is from multiple ESTs; TSL3—the only support is from a single EST; TSL4—the best supporting EST is flagged as the suspect; and TSL5—no single transcript supports the model structure. (**C**) Nuclear/cytoplasmic localization of *Kalnc1*, *3*, *4*, *6* and *7*. NRT; “No reverse transcriptase” control to rule out trace DNA contamination.

The full-length mRNA transcript (15.4 kb) arising from the mouse *Kalrn* gene consists of 60 exons spread over 604.4 kb of the genome (Figure 1) and matches the complexity of the human *Kalrn* gene. The start codon occurs within the first exon, and the stop codon of the largest isoform occurs in the 60th exon. Alternative splicing of these protein-coding transcripts gives rise to about 14 coding variants, of which Kal7, Kal9, and Kal12 have been characterized in relatively greater detail. Besides these coding transcripts, we found seven presumably non-coding transcripts, referred to here as *Kalnc1* to *Kalnc7*, all expressed in the same direction as the protein-coding transcripts. The protein-coding transcripts of the *Kalrn* gene are known to have at least four alternate start sites, often referred to as A, B, C, and D. Some of these transcripts comprise many exons and are several kilobases in length. For instance, *Kalnc1* consists of 37 exons collectively account for 6.1 kb in length after removal of introns. Although it overlaps significantly with the protein-coding transcripts (35/37 exons), the use of distinct exons at the termini, avoiding the canonical start codon, results in a low coding potential. Some other ncRNAs from the locus were as short as 373 nts, comprising only 2 exons. We next checked their coding potential and reliability of the annotation (Figure 1A). For coding potential analysis, we used the CPAT algorithm, which recommends a score of less than 0.44 for non-coding transcripts. *Kalnc2* and *Kalnc5* showed low coding potential scores of 0.15 and 0.09, respectively, whereas the other ncRNAs arising from the *Kalrn* locus have scores ranging from 0.75 to 1.00, perhaps attributable to the overlap with coding exons of the *Kalrn* gene. Four of the linear ncRNAs, *Kalnc1*, *Kalnc2*, *Kalnc3*, and *Kalnc6*, are bioinformatically assembled transcripts, while *Kalnc4* has a relatively better level of confidence, with every splice junction being supported by at least one transcript. Interestingly, we found that a 294 nts circular RNA, apparently formed by the back splicing of exons 53 and 54, has also been detected in high-throughput sequencing but has not been verified further [24,25]. Taken together, bioinformatics predictions suggested that the *Kalrn* gene locus gives rise to seven linear and one circular RNA (Figure 1B), calling for a thorough experimental validation that is essential for studying the regulatory potential of this region.

**Figure 2:**
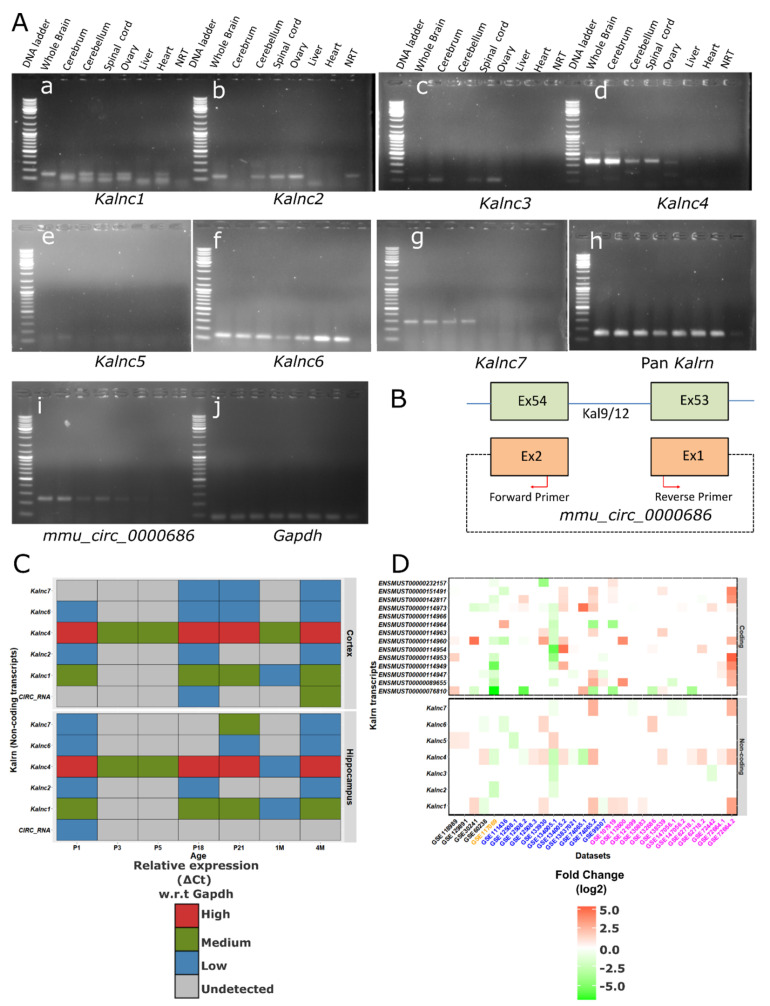
*Kalrn* locus lncRNAs are brain-enriched. (**A**) Representative images of endpoint RT-PCR showing expression of *Kalrn* loclncRNAs: *Kalnc1* (155 bps), *Kalnc2* (131 bps), *Kalnc3* (112 bps), *Kalnc4* (376 bps), *Kalnc5* (142 bps), *Kalnc6* (138 bps), *Kalnc7* (295 bps); pan *Kalrn* (157 bps), *mmu_circ_0000686* (217 bps), and *Gapdh* (75 bps) in (**a**–**j**) respectively in the whole brain, cerebrum, cerebellum, spinal cord, ovary, liver, and heart. (**B**) Schematic illustration of the circular RNA mmu_circ_0000686 at mouse *Kalrn* locus. (**C**) Relative expression of *Kalrn* locus lncRNAs was measured in mouse cortex and hippocampus at different time points (P1, P3, P5, P18, P21, 1 M, and 4 M) by qRT-PCR. (Data are shown as mean (SD), *N* = 3 biological replicates; *n* = 3 technical replicates). (**D**) A heatmap of mouse datasets associated with schizophrenia (black label), addiction (orange label), epilepsy (blue label), and autism (magenta label). Each row represents protein-coding (top panel) or non-coding (bottom panel) transcripts arising from the *Kalrn* locus. Labels include ID of GEO datasets.

Using total RNA isolated from the brain of adult mice and primers designed to differentiate between isoforms, we carried out RT-PCR to check the presence of these novel transcripts of unknown function. We also included a pair of pan-*Kalrn* primers that would detect the coding transcripts. As expected, the pan-*Kalrn* primers support the notion that *Kalrn* is expressed across tissues, and *Kalnc1*, the largest non-coding isoform, was also similar (Figure 2A). Even with this single pair of primers, it was obvious that *Kalnc1* shows further splicing heterogeneity. *Kalnc6*, a relatively small ncRNA, was also expressed in all the tissues tested. The predicted circular RNA [24,25] was detected using divergent PCR primers (Figure 2B). The size of the observed band was 217 nts, slightly smaller than the predicted 294 nts. It was readily detected in the cerebrum, cerebellum, spinal cord, and ovary of adult mice. During development, it was detected only in the hippocampus at postnatal day 1.

Our group has previously shown that in zebrafish, a lncRNA arising from the *kalrna* gene locus is maternally inherited [23]. To test if the mammalian locus also had potentially inherited lncRNAs, we included RNA from the mouse ovary in our RT-PCR experiments. All the ncRNAs barring *Kalnc7* showed expression in the ovary besides being expressed in one or more parts of the central nervous system. *Kalnc3* and *Kalnc4* were particularly interesting because they were specifically expressed in the cerebrum and cerebellum, respectively.

Since these transcripts overlap with coding transcripts and each other, in situ hybridization using larger probes cannot be used to resolve the spatial expression pattern in greater detail. To look at the brain expression pattern in greater detail, we carried out quantitative RT-PCR on RNA from the cortex and hippocampus in mice of postnatal day 1,3, 5 and 18 followed by 3 weeks, 1 month and 4 months. As shown in Figure 2B, *Kalnc4* was readily detectable at all developmental stages in the cortex and hippocampus. This level of abundance is higher than expected for lncRNAs since it roughly correlates to one or two orders of magnitude less than the abundant *Gapdh* mRNA. All the other lncRNAs were far less abundant, but a general trend in the expression pattern was that all the lncRNAs from this locus were least abundant at P3 and P5 (postnatal days 3 and 5), a period that coincides with the switch from neurogenesis to astrogenesis (Figure 2C). During the later stages, when synapses mature, the expression of the *Kalrn* locus lncRNAs is again relatively high.

Further, we analyzed 209 publicly available RNA-Seq datasets pertaining to the disease conditions that are already linked to the *Kalrn* locus (addiction-17, autism-120, schizophrenia-38, and epilepsy-34). The datasets were internally normalized, and fold changes were calculated with respect to corresponding control samples (see methods for details). We found *Kalnc4* is upregulated in several autism-related datasets, while it is downregulated in mice addicted to nicotine (Figure 2D). The largest lncRNA, *Kalnc1*, was upregulated in a number of conditions. Interestingly, *Kalnc2* resembled protein-coding isoforms of Kalirin in that it was downregulated in the nucleus accumbens of mice addicted to nicotine. Thus, lncRNAs arising from the *Kalrn* locus have specific developmental expression profiles and are differentially expressed in disease models.

**Figure 3:**
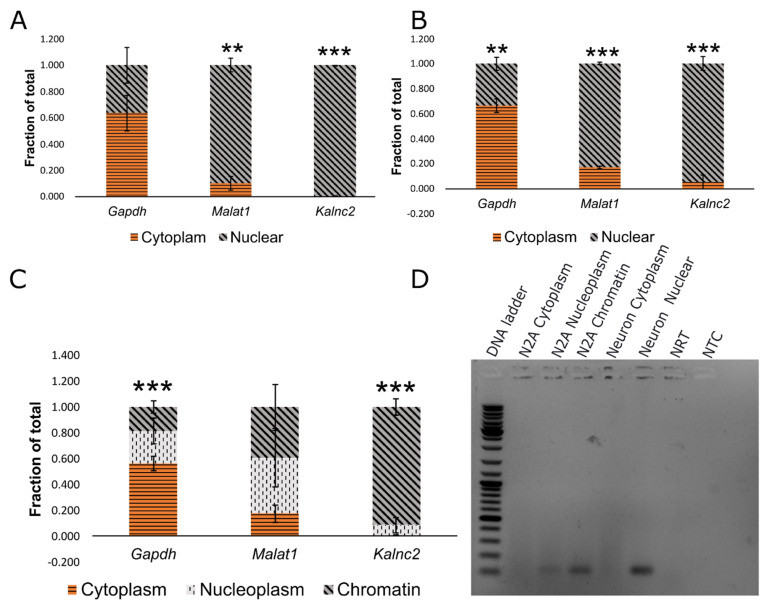
*Kalnc2* is enriched in the nuclear fraction. (**A**–**C**) *Kalnc2* expression was measured along with *Gapdh* (a cytoplasmic marker) and *Malat1* (a nuclear marker) in cellular fractions at (**A**) DIV0 (days in vitro culture), (**B**) DIV15 primary cortical neurons, and (**C**) N2A cells by qRT-PCR. (**D**) Endpoint PCR of *Kalnc2* in cellular fractions of N2A (Neuro2A) cells and primary cortical neurons. NRT (No reverse transcriptase control), NTC (No template control), and DIV (Days of in vitro culture). (Data are shown as mean ± SD; *N* = 3 biological replicates; *n* = 3 technical replicates; *t*-test, ** *p* < 0.01; *** *p* < 0.001).

*Kalnc2* was of particular interest because of its location of origin and its temporal expression pattern. As shown in Figure 1B, *Kalnc2* is a relatively small 523-nt lncRNA comprising 2 exons with a very low coding potential. Its location coincides with the 5′ end of the *Kalrn* protein coding isoforms, similar to the zebrafish durga lncRNA that we have previously identified. To understand the role of this lncRNA in greater detail, we studied its sub-cellular localization in primary cortical neurons in culture at the time of plating and at 15 days of culture (Figure 3A,B). As is the case with zebrafish *durga*, it was largely present in the nucleus. To resolve the intranuclear localization in further detail, we used the rodent neuroblastoma cell line Neuro2A. Here too, *Kalnc2* was localized to the nucleus, and further, it was enriched in the chromatin fraction (Figure 3C,D). *Malat1* and *Gapdh* showed the expected nuclear and cytoplasmic localization, respectively, ruling out the mixing of fractions during the experiment.

**Figure 4:**
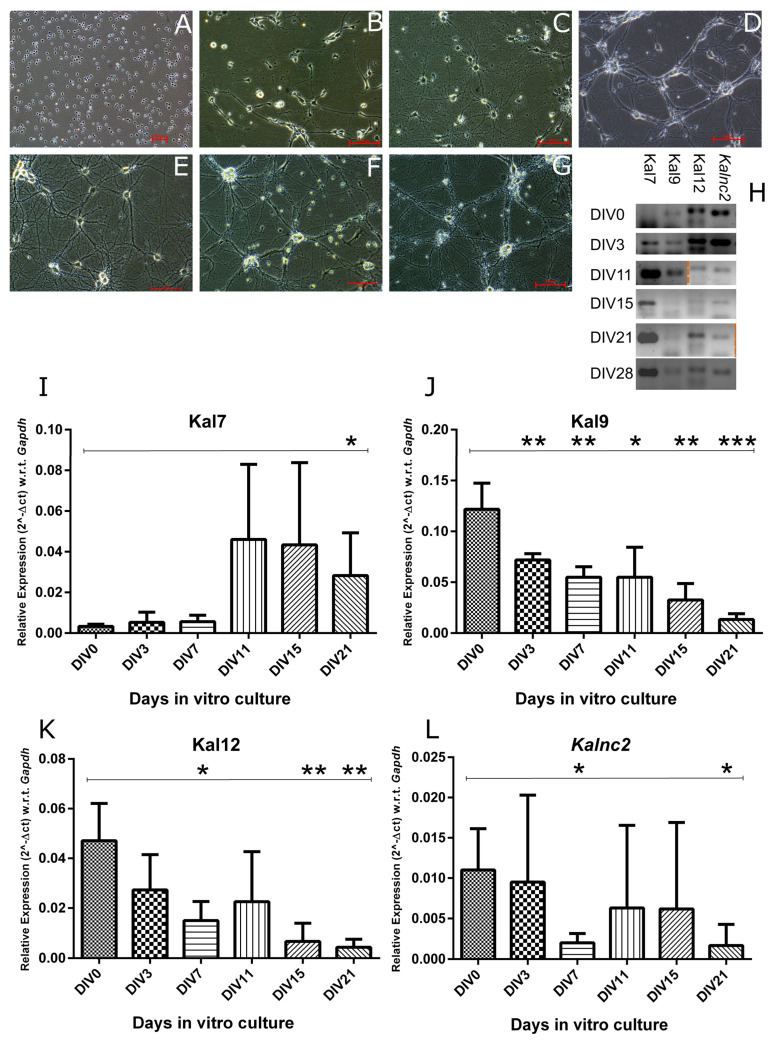
*Kalnc2* expression is similar to that of Kal9 and Kal12, but opposite to Kal7. (**A**–**G**) Bright field images of mouse neural stem cell differentiation and maturation in neurons. (**A**) DIV0, (**B**) DIV3, (**C**) DIV7, (**D**) DIV11, (**E**) DIV15, (**F**) DIV21, and (**G**) DIV28; scale bare 100 µm. (**H**) Endpoint PCR for *Kalnc2* (110 bps) and *Kalrn* major coding transcripts: Kal7 (127 bps), Kal9 (125 bp) and Kal12 (140 bps) at different DIVs (DIV0, DIV3, DIV11, DIV15, DIV21 and DIV28). (**I**–**L**) Kal7, Kal9, Kal12, and *Kalnc2* expressions were measured at DIV0, DIV3, DIV11, DIV15, and DIV21. DIV (Days of in vitro culture). (Data are shown as mean ± SD; *N* = 3 biological replicates; *n* = 3 technical replicates; *t*-test, * *p* < 0.05; ** *p* < 0.01; *** *p* < 0.001).

**Figure 5:**
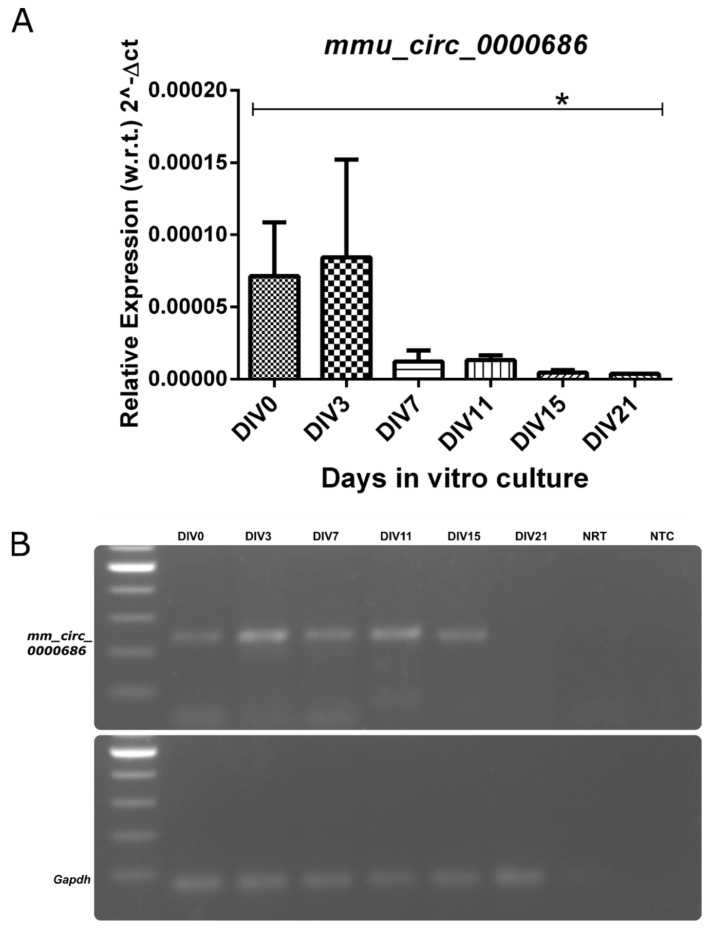
The expression of circular RNA decreases, as cortical neurons mature. (**A**,**B**) Circular RNA expression was measured at DIV0, DIV3, DIV7, DIV11, DIV15, and DIV21 with respect to *Gapdh* by quantitative RT-PCR. DIV (days of in vitro culture). (Data are shown as mean ± SD; *N* = 3 biological replicates; *n* = 3 technical replicates; *t*-test, * *p* < 0.05).

Next, we characterized the expression profiles of *Kalnc2* and the major *Kalrn* mRNA isoforms, Kal7, 9, and 12, during in vitro differentiation of cortical neurons (Figure 4). In agreement with previous reports, Kal9 and Kal12 are expressed during the early proliferative stages, but as neurons start forming synapses and these synapses mature, their expression is reduced. In sharp contrast, the expression of the Kal7 isoform, rises only in the later stages. In vivo, it has been shown that Kal9 and Kal12 dominate during embryonic development, while Kal7 is largely found in the adult brain. *Kalnc2* showed a strong similarity in expression pattern to Kal12, which is essential for axonal outgrowth. Like Kal12, it was expressed most strongly in the early stages of culture and decreased gradually with the formation of the neuronal network. The circular RNA arising from a back splicing of exon 54 and exon 53 of the *Kalrn* gene corresponding to Kal9/12 isoforms was also expressed highly in the initial stages but decreased during differentiation until it was undetectable at 21 days in vitro (Figure 5).

**Figure 6:**
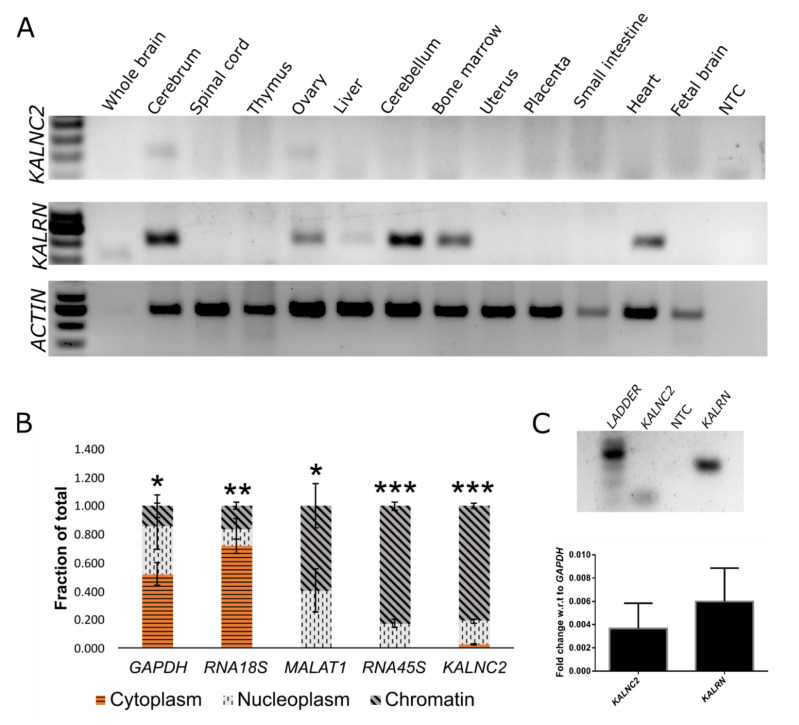
*hsKALNC2* is exclusively expressed in brain tissues. (**A**) Expression of *KALNC2* (119 bp) and *KALRN* (238 bp) in human tissue RNA panel, available commercially (**B**) Subcellular fractionation of SH-SY5Y cells using *RNA18S, MALAT1*, and *RNA45S* as cytoplasmic, nucleoplasmic, and chromatin markers, respectively. (**C**) Expression of *KALNC2* and *KALRN* in SH-SY5Y cells normalized to *GAPDH* expression. (Data are shown as mean ± SD; *N* = 3 biological replicates; *n* = 3 technical replicates; *t*-test, * *p* < 0.05; ** *p* < 0.01; *** *p* < 0.001).

In parallel, we explored the role of a lncRNA arising from a comparable location in the *KALRN* locus of the human genome. The human *KALRN* locus is comparable to the mouse locus in complexity, with 29 ncRNAs arising from the 692 kb locus. We found a transcript (ENST00000684441.1/ENST00000488825.5) (supplementary data Figure S1) that was annotated in the direction of the coding mRNAs at the 5′ end—a position corresponding to the *durga* lncRNA in zebrafish and *Kalnc2* in mouse. Since the first exon of this transcript showed sequence conservation across vertebrates, this lncRNA is henceforth referred to as *hsKALNC2* (supplementary data Figure S2). We used directional reverse transcriptase PCR to validate the expression and confirm the orientation of this lncRNA. In agreement with the observation in mice, this RNA is also expressed in the cerebrum and ovary, but is excluded from tissues like cerebellum, liver, and heart where *Kalrn* mRNA is readily detected (Figure 6A). In human tissues and in the cell line SH-SY5Y, the lncRNA was of much lower abundance when compared to *KALRN* mRNA transcripts (Figure 6A). The *hsKALNC2* transcript was closely associated with the chromatin fraction (Figure 6B) in SH-SY5Y cells where it was expressed along with *KALRN* mRNA (Figure 6C). Taken together, we propose that mammalian genomes express a syntenically conserved lncRNA from the 5′ end of the *Kalrn* protein-coding gene, which is nuclear localized and selectively enriched in specific brain and ovary regions. The *mmKalnc2* and *hsKALNC2* genes share an identical 200-nts stretch at the 5′ end suggesting that mutational studies in the future should focus on this region. Owing to their conservation in mammals and relative synteny with respect to zebrafish *durga* lncRNA, we reasoned that they may be involved in similar functions.

**Figure 7:**
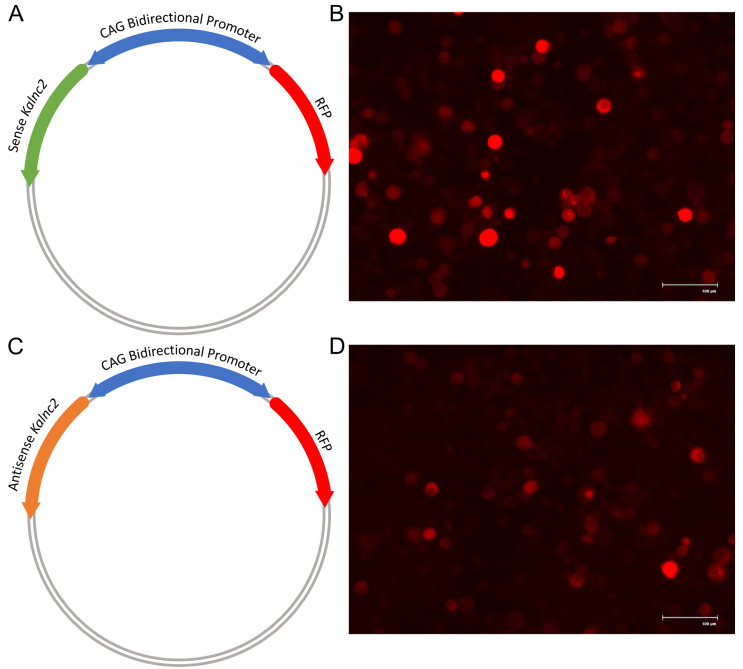

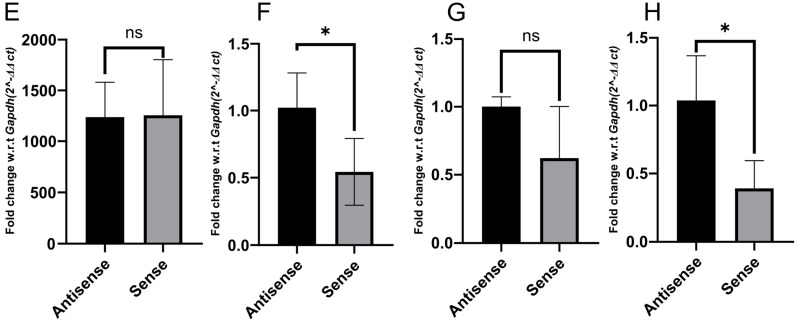
*Kalnc2* overexpression in N2A cells downregulates major *Kalrn* transcript expression. (**A**,**C**) A schematic of a bidirectional promoter plasmid containing *Kalnc2* and an RFP tag. (**B**,**D**) *Kalnc2* sense and antisense overexpressed RFP-positive cells, respectively. (**E**–**H**) Expression of *Kalnc2*, Kal7, Kal9, and Kal12, respectively, normalized by *Gapdh* in sense and antisense conditions (Data are shown as mean ± SD; *N* = 3 biological replicates; *n* = 3 technical replicates; *t*-test, * *p* < 0.05).

To test for a direct regulatory role of lncRNA in the expression of *Kalrn* mRNAs, we modulated the expression level of *mmKalnc2*. The *mmKalnc2*-specific transcript was expressed in sense and antisense orientation (as a control) under a bidirectional promoter such that transfected cells would coexpress a Red Fluorescent Protein (RFP) allowing their visualization. This clone was transfected into Neuro2A cells using lipofectamine (see materials and methods for details), and total RNA was collected 48 h post-transfection. We measured the steady-state expression of mouse Kal7, 9, and 12 using isoform-specific primers [27] in qRT-PCR. We could change the expression level of the lncRNA by >100 fold, irrespective of the orientation (Figure 7E). As shown in Figure 7B,D when *Kalnc2* (sense transcript) was overexpressed, we find that Kal7, Kal9 and Kal12 mRNAs were downregulated by about 50% (Figure 7F–H).

## 3. Discussion

Our results reveal that the *Kalrn* locus gives rise to several linear and one circular ncRNA that are selectively expressed in the adult mouse nervous system. The circular RNA, *mmu_circ_0000686*, *mmKalnc2*, and *hsKALNC2* from the human *Kalrn* gene locus are also expressed during the early stages of development. The same pattern is recapitulated during in vitro differentiation, showing a gradual decrease with time.

The circular RNA, *mmu_circ_0000686,* and the linear RNAs, *Kalnc2* and *Kalnc5*, were clearly non-coding. The other RNAs described here showed some coding potential in our bioinformatics analysis but were exclusively localized to the nucleus. In our opinion, the ambiguity in the coding potential of *Kalnc1, 3, 4*, *and 7* is due to the overlap with protein-coding RNAs. The inclusion of introns, which usually contain stop codons, may convert a coding transcript into a non-coding variant. *Kalnc6* showed some coding potential and is at least partially localized to the cytoplasm. Further studies are required to verify the non-coding nature of this transcript. The ultimate evidence for the coding status of a transcript can only be provided by the demonstration of the protein product. Association with ribosomes and the presence of short ORFs are both now known to be only indicators of coding potential [28]. Further, certain transcripts may perform non-coding RNA functions and also give rise to small peptides or proteins [29].

Compared to mRNAs, lncRNAs are usually much lower abundance. Among the non-coding RNAs arising from the *Kalrn* gene locus, only *Kalnc4* is expressed at high levels, comparable to the relatively abundant mRNA *Gapdh*. All the other lncRNAs are expressed at a baseline about two orders of magnitude lesser than *Kalnc4*. However, these RNAs are transiently induced several folds in the mouse cortex around postnatal day 18 but are barely detectable in the 1-month-old mouse brain. All the non-coding RNAs are again readily detectable in the 4-month-old mouse brain. Since mouse puberty occurs at around 38 days, we speculate that the non-coding RNAs of the *Kalrn* locus may be induced during puberty, besides a transient spike in expression during development.

The mammalian *Kalrn* gene locus has been extensively mined for mutations associated with several neurodevelopmental and neuropsychiatric diseases [9,10,11]. Meta-analysis of gene expression data reported by various groups studying mouse models of these diseases showed that *Kalnc1*, *2*, *4*, and *7* show altered expression in models of autism and nicotine addiction. The protein-coding isoforms arising from the locus have several interaction domains that provide spatiotemporal heterogeneity to the core Rho GTPase role of the Kalirin protein. Many protein-coding isoforms depend on different promoters for their expression, while the relationship between regulatory elements and non-coding RNAs has not been explored. Certain isoforms promote axonal outgrowth, while others determine dendrite numbers, dendritic arborization, and the maturation of dendritic spines. As neurons mature, the expression of the longer protein-coding isoforms, Kal9 and Kal12, decreases with a concomitant increase in Kal7. We used overexpression of *Kalnc2* to explore the possibility that it regulates the expression of protein-coding *Kalrn* transcripts. When we ectopically overexpressed it in N2A cells, we found Kal7, Kal9, and Kal12 mRNAs were downregulated. However, it appears that *Kalnc2* may have no effect on the switch from longer to shorter *Kalrn* protein-coding isoforms during neuron differentiation and maturation.

There are several caveats to unequivocally establishing the function of *Kalnc2*. More refined experiments addressing the role of *Kalnc2* in specific neuronal subtypes or in specific regions of the brain may reveal a regulatory role for these lncRNAs in vivo. Loss of function studies through knock-down and mutations are needed to establish the function of this lncRNA. We cannot rule out the possibility that localization and RNA modification of endogenous *Kalnc2* may not be replicated when overexpressed ectopically.

In spite of the apparent lack of sequence conservation, the lncRNAs *durga* (zebrafish), *mmKalnc2*, and *hsKALNC2* show striking similarities: they share the same protein-coding neighbor, associate with chromatin, and are expressed in immature neurons. In the future, chromatin immunoprecipitation can reveal the genomic region to which these lncRNAs bind, perhaps in association with chromatin remodeling complexes. They may also be involved in defining 3D chromatin organization. Neuronal differentiation and maturation are accompanied by large-scale changes in 3D chromatin structure. It will be interesting to study the role of lncRNAs, especially those from the *Kalrn* locus, that could contribute to such spatial reorganization of chromatin in the nuclei of neurons during differentiation. Regulatory RNAs inherited through the maternal ooplasm are a form of epigenetic inheritance [30]. Recent studies have shown that gametes are a rich source of regulatory RNAs, including miRNA [30], tRNA fragments [31], and lncRNA [32]. A lncRNA arising from the locus is inherited through the maternal cytoplasm of oocytes in zebrafish [23]. The expression pattern of the mammalian lncRNAs raises the possibility that they are also inherited, although it is premature to assume ovarian expression corresponds to expression in oocytes since we cannot rule out the possibility that the RNA is localized to the non-gametic tissue of the ovary. In summary, we report that several, hitherto unknown, non-coding RNAs are dynamically expressed from the mammalian *Kalrn* locus, and one such RNA, *Kalnc2*, can downregulate major *Kalrn* protein-coding transcripts, highlighting the regulatory potential of this genomic region that is implicated in several neuropsychiatric and neurodevelopmental disease conditions.

## 4. Materials and Methods

### 4.1. Genome and Transcriptome Meta-Analysis

All the analysis and visualization for the mouse genome was carried out on (GRCm38/mm10), and similarly, the human genome (GRCh38/hg38) was used. The *KALRN* genomic locus (mouse = chr16: 33,969,073-4,573,532; human = chr3: 124,033,369-124,726,325) was accessed with the tracks from Gencode v.38 (Ensembl 104) or Gencode v.M25 for mouse (Ensembl 100). Treating *Kalrn*/ENST00000682506.1 as the longest transcript, all transcripts within its limits were then further analyzed. Five transcripts that were mentioned as “processed transcripts” and two that denoted “intron-retained” were observed. These transcript sequences were downloaded and used for coding potential analysis in the CPAT software [26] (http://lilab.research.bcm.edu/) (accessed on 30 June 2022). Oligonucleotide primers (Supplementary Data Table S1) specific for transcripts and pan-*Kalrn* were designed using Primer 3.

### 4.2. Animals

All experimental procedures were performed on Balb/c mice and approved by the Institutional Animal Ethics Committee of the CSIR-Institute of Genomics and Integrative Biology (IGIB) and followed appropriate guidelines for live animal use in research. All mice used in this study were bred in the CSIR-IGIB animal house facility. They were kept at a temperature of 24 ± 2 °C on a 12 h light/dark cycle, with ad libitum access to food and water. All institutional guidelines were followed for animal handling and experiments.

### 4.3. Primary Cortical Neuron Culture

Adult male and female mice were kept for mating at a 1:2 ratio, and pregnancy was confirmed by checking the vaginal plug, and after a few days of mating, it was re-confirmed by measuring the weight of a female. Pregnant females were anesthetized with thiopentone (40 mg/kg) at Embryonic days 16–8 (E16-18). The embryos were dissected out by making a straight incision in the abdomen. Embryos were immediately kept in cold HBBS (14025 Gibco, Billings, MT, USA), and brains were removed. Carefully, meninges and blood vessels were removed, and cortices were dissected out. They were transferred into the 15 mL tube containing cold HBSS. Cortices were washed three times with HBSS for 5 min at 500 g and 0.25% trypsin-EDTA (25200-0560, Gibco) was added, then triturated by a Pasteur pipette and kept at 37 °C for 5 min. Trypsin-EDTA was inactivated by (10082-147, Gibco), and tissue was centrifuged at 500× *g* for 5 min, then washed twice with HBSS, and a single cell suspension was made in complete neurobasal media (Neurobasal; 10888-022, Gibco + B27; A35828-01, Gibco + Glutamax; 35050-061, Gibco + Primocin; ant-pm-1, Invivogen, Waltham, MA, USA). Cells were counted and seeded on the poly-D lysine (P7405, Sigma, St. Louis, MO, USA) coated (0.1 mg/mL solution) plates. The first media change, replacing half of the medium, was done after 12 h of cell seeding, and thereafter twice a week. Cells were collected at different days in vitro culture (DIV): DIV0 (the day when cells were seeded), DIV3, DIV7, DIV11, DIV15, DIV21, and DIV28.

### 4.4. Neuro2A Cell Culture

N2A cells (originally sourced from the National Cell Repository) were cultured in Dulbecco’s Modified Eagle’s Medium (DMEM, Invitrogen) supplemented with 10% fetal bovine serum (FBS) and incubated in a humidified incubator with 5% CO_2_.

### 4.5. SH-SY5Y Differentiation

SH-SY5Y cells (source: European Collection of Authenticated Cell Cultures) were cultured in Dulbecco’s Modified Eagle’s Medium (DMEM, Invitrogen) supplemented with 10% fetal bovine serum (FBS) and incubated in a humidified incubator with 5% CO_2_. For differentiation of SH-SY5Y, cells at passage number 30–33 were treated with 10 µm of *trans* retinoic acid (RA) at day 1, cells were then grown for 4 days with media change at every alternative day and replaced with 10 ng/mL BDNF and 10µm RA at day5 and grown for 10 days. At day 10, DMEM was replaced with neurobasal media, and cells were collected at day 12.

### 4.6. Kalnc2 over-Expression

A *Kalnc2*-specific fragment of 321 nts (chr16:34,512,74034,513,074) and reporter mCherry were cloned under the mammalian CAG-bidirectional promoter and transfected by lipofectamine 2000 (116668-019, Invitrogen) into the Neuro2A cells. After transfection, cells were collected at different time points, and the expression of *Kalnc2* along with *Kalrn* protein-coding transcripts was measured by quantitative RT-PCR.

### 4.7. RNA Isolation and RT-PCR

Mice were euthanized with sodium thiopental (40 mg/kg), and the brain and other body parts were removed. RNA was isolated using trizol (15596018, Ambion, Austin, TX, USA) from the whole brain, cortex, hippocampus, cerebellum, ovary, liver, heart, and primary cortical neurons. cDNA was prepared from 1 µg of total RNA, and then RT-PCR was done using the SYBR Green master mix (PKG025-A Genetix). *Gapdh* was used as a normalization control in RT-PCR data analysis. The RT-PCR products were visualized by agarose (2%) gel electrophoresis.

### 4.8. Subcellular Fractionation

Cells were washed with 1×PBS and then collected with 0.25% trypsin-EDTA, centrifuged (100–200× *g* for 5 min), and counted. A 175 µL cytoplasmic lysis buffer (tris-HCl pH 8.0 50 mM, NaCl 140 mM, MgCl2 1.5 mM, NP-40 0.5%, DTT 1 mM, EDTA 5 mM) was added to 1 × 10^6^ cells, suspended, and incubated at ice for 5 min, and then cell suspension was centrifuged at 300× *g* for mins at 4 °C. The supernatant was transferred into the fresh tube, and it is a cytoplasmic fraction. The pellet was washed twice with 200 µL of cytoplasmic lysis buffer at 300 g for 2 min at 4 °C. 175 µL nuclear lysis buffer (Tris HCl pH 8.0, 50 mM, NaCl 500 mM, MgCl2 1.5 mM, NP-40 0.5%, and DTT 1 mM) were added per 1 × 10^6^ cells pellet, suspended, and incubated at ice for 5 min. The cell suspension was then centrifuged at 16,400× *g* for 2 min at 4 °C. The supernatant was collected which constituted soluble nuclear fraction, and the pellet corresponding to the chromatin fraction, were separated, and the RNA isolation, cDNA preparation, and RT-PCR were done for all three fractions. The RT-PCR products were visualized by agarose (2%) gel electrophoresis. Subcellular fractions were confirmed by respective fraction markers: *Gapdh* and *RNA18S* (cytoplasmic markers), *Malat1* (nuclear markers), and *RNA45S* (chromatin markers), since 45S rRNA is chromosome-associated [33].

### 4.9. Meta-Analysis of Expression Datasets

The 209 datasets associated with the query (addiction OR autism OR epilepsy OR schizophrenia) AND “Mus musculus” [porgn] AND (“Expression profiling by high-throughput sequencing” [Filter]) were downloaded from Gene Expression Omnibus (GEO). Raw SRA sample files were converted into fastq format by fastq-dump [34], adapter trimming was performed by trimmometic [34], and finally, quality control was done by fastqc [35] software. Passed quality control samples were aligned on mouse assembly (GRCm38/mm10|: Chr16) by STAR [36], reads were counted by RSEM [37], and differential expression and fold change were calculated by EBSeq [36].

## Data Availability

All data generated or analysed during this study are included in this published article and its Supplementary Materials files.

## Supplementary Materials

The following supporting information can be downloaded at: https://www.mdpi.com/article/10.3390/ncrna9010007/s1, Figure S1: View of the *Kalrn* gene locus of humans, mice, and zebrafish, taken from the UCSC genome browser; Figure S2: Sequence alignment of *mmKalnc2* and hsKALNC2. NCBI BLAST was used for the alignment, *mmKalnc2* was the subject, and hsKALNC2 was the query; Table S1: Primer list.
