# Supplementary material for: A Novel Cis-Regulatory lncRNA, Kalnc2, Downregulates Kalrn Protein-Coding Transcripts in Mouse Neuronal Cells
Source: Noncoding RNA. 2023 Jan 13;9(1):7. doi: 10.3390/ncrna9010007 (PMC9844340; doi:10.3390/ncrna9010007)
Supplement: Supplementary file 1 [file ncrna-09-00007-s001.zip › ncrna-2062159-supplementary.pdf]

Supplementary information

Table S1: Primer list

**Primer name**	**Sequence**
mmKalnc1_RP	CACAAAGGCCACTTTCTCCT
mmKalnc1_FP	CTCCCCCAGTCATGAACC
mmKalnc2_FP	TCGGGAGAAATCAAAGATGG
mmKalnc2_RP	GGATCCATTCTCCATCCTCA
mmKalnc3_FP	TGGGTCAGATGAACAGAGCA
mmKalnc3_RP	GAGGAGGGGCTTGATGAGAT
mmKalnc4.1_FP	GTCAACGCCAGCTCTTTGTC
mmKalnc4.1_FP	CCTGGCTTCTGATGCTGTACTA
mmKalnc4.2_RP	TGTTACAGTGGGAGTCTGATGG
mmKalnc4.2_FP	ATGCTTGGAATGGAAACTGTCT
mmKalnc5_FP	AAGCTGGACATCTTCCTACAGC
mmKalnc5_RP	GAACGACTCAGGTTCCATTGTC
mmKalnc6_FP	GTGGAGAACTCGGGTAAAAATG
mmKalnc6_RP	AGTGATTTTCATTGTCCCAAGG
mmKalnc7_FP	TGTGTTGCAATACCAAGCTTTA
mmKalnc7_RP	GAGCTTCACTCGAGGCACTG
mouse Kalirin-7(Kal7)_FP	GATACCATATCCATTGCCTCCAGGACC
mouse Kalirin-7(Kal7)_RP	CCAGGCTGCGCGCTAAACGTAAG
mouse Kalirin-9(Kal9)_FP	GCCCCTCGCCAAAGCCACAGC
mouse Kalirin-9(Kal9)_RP	CCAGTGAGTCCCGTGGTGGGC
mouse Kalirin-12(Kal12)_FP	CAGCAGCCACGTGCCTGCAGC
mouse Kalirin-12(Kal2)_RP	TCTTGACATTGGGAATGGGCCGCAC
mouse full-length Kalirin_FP	GCCTTTCTCAGCAAACACACTGGGG
mouse full-length Kalirin_RP	ATTCCCCAGTCTGAGCCAGCTGC
mmGapdh_FP	CTACACTGAGGACCAGGTTGTCT

mmGapdh_FP	TCATACCAGGAAATGAGCTTGAC
mmMalat1_FP	GGCGGAATTGCTGGTAGTTT
mmMalat1_RP	AGCATAGCAGTACACGCCTT
mm_circ_000686_FP	AGTGGGATTTATACCTGCATAGC
mm_circ_000686_RP	GGAGGAGATGGTGTATGTTGC
huKALNC2_ FP	GTGTGGTCCTGGTGAGGATG
huKALNC2RP	CCCATGATGGAGATGACACC
human Kalirin_FP	CATGGTGAGGCCTTTCTCAGCAAAC
human Kalirin RP	CAGGTGTCGAGCTGCCTTGTAGAT
huGAPDH_FP	GGGAGCCAAAAGGGTCATCA
huGAPDH_RP	TAAGCAGTTGGTGGTGCAGG
humalat1_FP	CGCTTGAGATTTGGGCTTTA
humalat1_RP	CTTCCTGTGGCAGGAGAGAC
hu18SrRNA_FP	GTAACCCGTTGAACCCCATT
hu18SrRNA_RP	CCATCCAATCGGTAGTAGCG
hu45SrRNA_FP	TCGCTGCGATCTATTGAAAG
hu45SrRNA_RP	AGGAAGACGAACGGAAGGAC
huTUBB3_FP	GCTCAGGGGCCTTTGGACATCTCTT
huTUBB3_RP	TTTTCACACTCCTTCCGCACCACATC
huGFAP_FP	CCTCTCCCTGGCTCGAATGC
huGFAP_RP	GGAAGCGAACCTTCTCGATGTA
huSOX2_FP	CATGTCCCAGCACTACCAGA
huSOX2_RP	TACCGGGTTTTCTCCATGCT
huNESTIN_FP	TCTTTGCTCCCAGTCCTGAG
huNESTIN_RP	GGGCTCTGATCTCTGCATCT
huMAP2_FP	CTGCTTTACAGGGTAGCACAA
huMAP2_RP	TTGAGTATGGCAAACGGTCTG

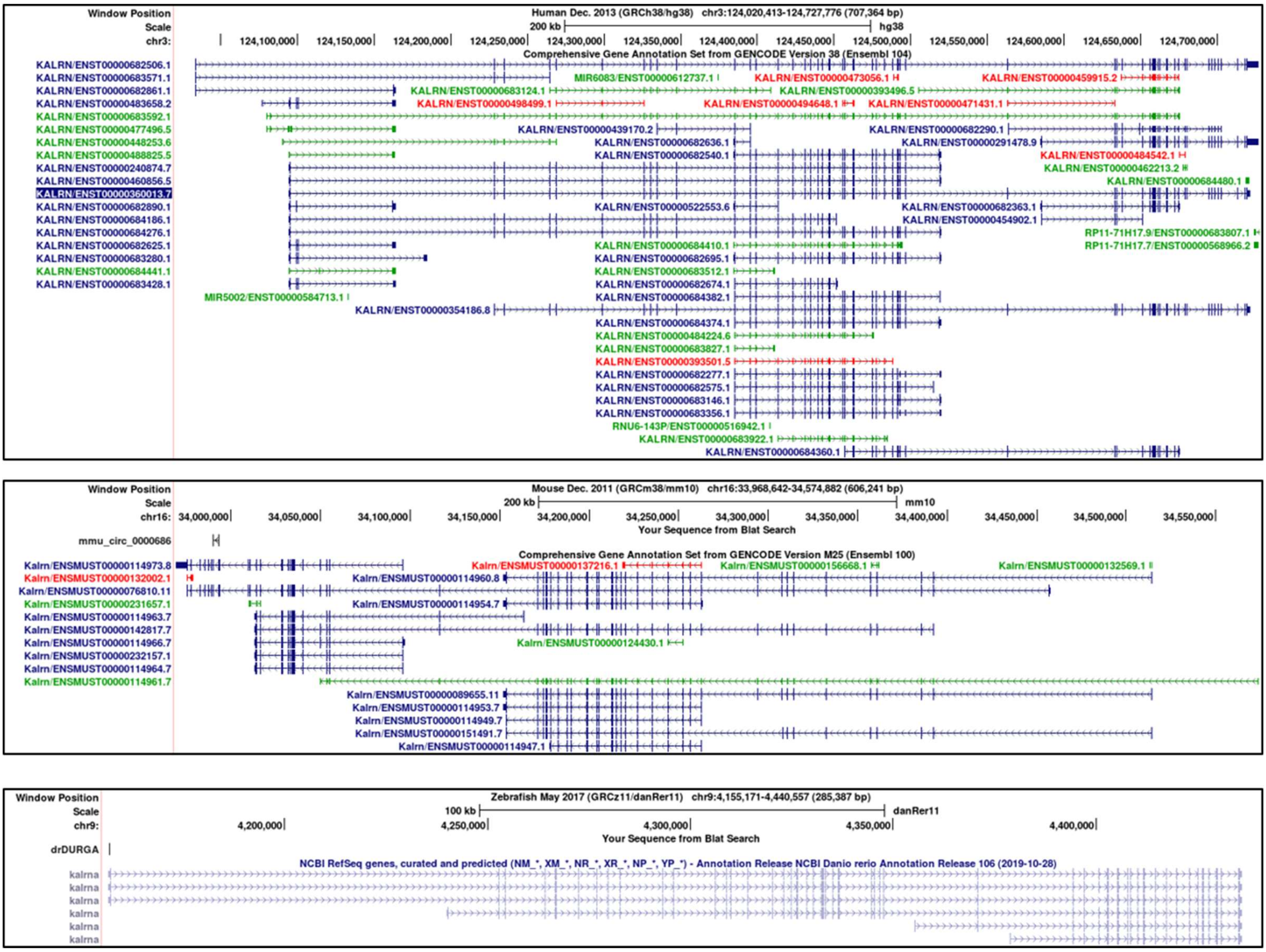

Figure S1. View of the Kalrn gene locus of human, mouse and zebrafish, taken from UCSC genome browser.

Query_45086:0..523 mmKalnc2(ENSMUST00000132569.2)

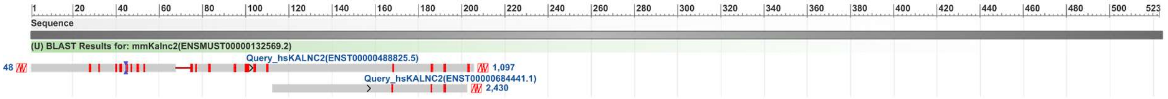

hsKALNC2(ENST00000488825.5)

Sequence ID: **Query_45089** Length: **1344** Number of Matches: **1**

Range 1: 49 to 247 Graphics ▼ Next Match ▲ Previous Match

Score	Expect	Identities	Gaps	Strand
210 bits(132)	7e-58	178/206(86%)	8/206(3%)	Plus/Plus

```
Query  1    AGGCAGGCATTTGCTTAGAGCAGGCTGCGTGTGAGCCCAACATC-CGAGAATCTGGCCTC  59
            ||||||||||||||||||||||||||| ||| ||||||| | ||  | || || ||||||
Sbjct  49   AGGCAGGCATTTGCTTAGAGCAGGCTGTGTGCGAGCCCAGCGTCAAGTGATTCCGGCCTC  108

Query  60   CTCGAGTCGGTTGGTGGTGGTGGCGGGATGAGGCTGTGCCCTGGAGACTGACTGTGAAGG  119
            ||||||||        | ||||| ||||||||||| ||||  || ||||| |||||||||
Sbjct  109  CTCGAGTC-------AGCGGTGGTGGGATGAGGCTCTGCCGAGGGGACTGGCTGTGAAGG  161

Query  120  ATGAGTTCAGGGTGGGATGACGGACCGCTTCTGGGACCAGTGGTATCTTTGGTATCTCCG  179
            |||||||||||||||||||||||||||||||||||||||||||||||| |||||||||||
Sbjct  162  ATGAGTTCAGGGTGGGATGACGGACCGCTTCTGGGACCAGTGGTATCTCTGGTATCTCCG  221

Query  180  CTTGCTTCGGCTTCTGGATCGAGCTT  205
            |||||| ||||| |||||||||| ||
Sbjct  222  CTTGCTCCGGCTGCTGGATCGAGGTT  247
```

hsKALNC2(ENST00000684441.1)

Sequence ID: **Query_45088** Length: **2520** Number of Matches: **1**

Range 1: 1 to 90 Graphics ▼ Next Match ▲ Previous Match

Score	Expect	Identities	Gaps	Strand
134 bits(84)	5e-35	87/90(97%)	0/90(0%)	Plus/Plus

```
Query  113  GTGAAGGATGAGTTCAGGGTGGGATGACGGACCGCTTCTGGGACCAGTGGTATCTTTGGT  172
            ||||||||||||||||||||||||||||||||||||||||||||||||||||||| ||||
Sbjct  1    GTGAAGGATGAGTTCAGGGTGGGATGACGGACCGCTTCTGGGACCAGTGGTATCTCTGGT  60

Query  173  ATCTCCGCTTGCTTCGGCTTCTGGATCGAG  202
            ||||||||||||| ||||| ||||||||||
Sbjct  61   ATCTCCGCTTGCTCCGGCTGCTGGATCGAG  90
```

Figure S2. Sequence alignment of *mmKalnc2* and *hsKALNC2*. NCBI blast was used for the alignment, *mmKalnc2* was the subject and *hsKALNC2* was the query.
